# RETRACTION: Comparative Study of Wound Outcomes and Surgical Strategies: Internal Fixation Versus External Stabilization in Rib Fracture Patients with Traumatic Chest Wounds

**DOI:** 10.1111/iwj.70613

**Published:** 2025-04-21

## Abstract

**RETRACTION:** WangD. , WangX. , WangQ. , XuYu. , and XuYo. , “Comparative Study of Wound Outcomes and Surgical Strategies: Internal Fixation Versus External Stabilization in Rib Fracture Patients with Traumatic Chest Wounds,” International Wound Journal 21, no. 4 (2023): e14548, 10.1111/iwj.14548.38151911 PMC10961044

The above article, published online on 27 December 2023, in Wiley Online Library (http://onlinelibrary.wiley.com/), has been retracted by agreement between the journal Editor in Chief, Professor Keith Harding; and John Wiley & Sons Ltd. Following an investigation by the publisher, all parties have concluded that this article was accepted solely on the basis of a compromised peer review process. In addition, further investigation by the publisher found that some statements received inadequate citations and that there were insufficient methodological details to assess the suitability of fracture treatment methods and the allocation of subjects between the two groups. The editors have therefore decided to retract the article. The authors did not respond to our notice regarding the retraction.

**RETRACTION:** D. Wang , X. Wang , Q. Wang , Yu. Xu , and Yo. Xu , “Comparative Study of Wound Outcomes and Surgical Strategies: Internal Fixation Versus External Stabilization in Rib Fracture Patients with Traumatic Chest Wounds,” International Wound Journal 21, no. 4 (2023): e14548, 10.1111/iwj.14548.38151911 PMC10961044

The above article, published online on 27 December 2023, in Wiley Online Library (http://onlinelibrary.wiley.com/), has been retracted by agreement between the journal Editor in Chief, Professor Keith Harding; and John Wiley & Sons Ltd. Following an investigation by the publisher, all parties have concluded that this article was accepted solely on the basis of a compromised peer review process. In addition, further investigation by the publisher found that some statements received inadequate citations and that there were insufficient methodological details to assess the suitability of fracture treatment methods and the allocation of subjects between the two groups. The editors have therefore decided to retract the article. The authors did not respond to our notice regarding the retraction.

